# Extend the benchmarking indel set by manual review using the individual cell line sequencing data from the Sequencing Quality Control 2 (SEQC2) project

**DOI:** 10.1038/s41598-024-57439-7

**Published:** 2024-03-25

**Authors:** Binsheng Gong, Dan Li, Yifan Zhang, Rebecca Kusko, Samir Lababidi, Zehui Cao, Mingyang Chen, Ning Chen, Qiaochu Chen, Qingwang Chen, Jiacheng Dai, Qiang Gan, Yuechen Gao, Mingkun Guo, Gunjan Hariani, Yujie He, Wanwan Hou, He Jiang, Garima Kushwaha, Jian-Liang Li, Jianying Li, Yulan Li, Liang-Chun Liu, Ruimei Liu, Shiming Liu, Edwin Meriaux, Mengqing Mo, Mathew Moore, Tyler J. Moss, Quanne Niu, Ananddeep Patel, Luyao Ren, Nedda F. Saremi, Erfei Shang, Jun Shang, Ping Song, Siqi Sun, Brent J. Urban, Danke Wang, Shangzi Wang, Zhining Wen, Xiangyi Xiong, Jingcheng Yang, Lihui Yin, Chao Zhang, Ruolan Zhang, Ambica Bhandari, Wanshi Cai, Agda Karina Eterovic, Dalila B. Megherbi, Tieliu Shi, Chen Suo, Ying Yu, Yuanting Zheng, Natalia Novoradovskaya, Renee L. Sears, Leming Shi, Wendell Jones, Weida Tong, Joshua Xu

**Affiliations:** 1 Division of Bioinformatics and Biostatistics, National Center for Toxicological Research, U.S. Food and Drug Administration, Jefferson, AR 72079 USA; 2 Cellino Bio, 750 Main Street, Cambridge, MA 02143 USA; 3 Office of Data Analytics and Research, Office of Digital Transformation, Office of the Commissioner, U.S. Food and Drug Administration, Silver Spring, MD 20993 USA; 4 State Key Laboratory of Genetic Engineering, School of Life Sciences and Human Phenome Institute, Fudan University, Shanghai, 200438 China; 5 Human Phenome Institute, Fudan University, Shanghai, 201203 China; 6 iGeneTech Bioscience Co., Ltd., 8 Shengmingyuan Rd., Changping, Beijing, China; 7 Clinical Diagnostics Division, Thermo Fisher Scientific, 46500 Kato Rd., Fremont, CA 94538 USA; 8 College of Chemistry, Sichuan University, Chengdu, 610064 Sichuan China; 9 Q squared Solutions Genomics, 2400 Ellis Road, Durham, NC 27703 USA; 10 Guardant Health, Inc., 505 Penobscot Drive, Redwood City, CA 94063 USA; 11 Integrative Bioinformatics Support Group, National Institute of Environmental Health Sciences, National Institutes of Health, Research Triangle Park, NC 27709 USA; 12 College of Life Sciences, Shanghai Normal University, Shanghai, 200234 China; 13 Center for Bioinformatics and Computational Biology, and the Institute of Biomedical Sciences, School of Life Sciences, East China Normal University, Shanghai, 200241 China; 14 CMINDS Research Center, University of Massachusetts, Lowell, MA 01854 USA; 15 Department of Epidemiology, School of Public Health, Fudan University, Shanghai, 200032 China; 16 ResearchDx, Irvine, CA 92618 USA; 17 Eurofins Viracor, LLC, 18000 W 99th St., Lenexa, KS 66219 USA; 18 Eurofins Viracor Biopharma Services, Inc., 18000 W 99th St., Lenexa, KS 66219 USA; 19 Agilent Technologies, Inc., 11011 N Torrey Pines Rd., La Jolla, CA 92037 USA; 20 Cancer Genomics Laboratory, Department of Genomic Medicine, MD Anderson Cancer Center, Houston, TX 77030 USA; 21 State Key Laboratory of Genetic Engineering and Collaborative Innovation Center for Genetics and Development, School of Life Sciences, Fudan University, Shanghai, 200438 China; 22 PathGroup, Nashville, TN 37217 USA; 23 Velsera, 6 Cityplace Dr Suite 550, Creve Coeur, MO 63141 USA

**Keywords:** Indel, Precision medicine, Bioinformatics, Quality control, Benchmarking, Cancer genomics, Sequencing, Computational biology and bioinformatics, Bioinformatics, Sequencing

## Abstract

Accurate indel calling plays an important role in precision medicine. A benchmarking indel set is essential for thoroughly evaluating the indel calling performance of bioinformatics pipelines. A reference sample with a set of known-positive variants was developed in the FDA-led Sequencing Quality Control Phase 2 (SEQC2) project, but the known indels in the known-positive set were limited. This project sought to provide an enriched set of known indels that would be more translationally relevant by focusing on additional cancer related regions. A thorough manual review process completed by 42 reviewers, two advisors, and a judging panel of three researchers significantly enriched the known indel set by an additional 516 indels. The extended benchmarking indel set has a large range of variant allele frequencies (VAFs), with 87% of them having a VAF below 20% in reference Sample A. The reference Sample A and the indel set can be used for comprehensive benchmarking of indel calling across a wider range of VAF values in the lower range. Indel length was also variable, but the majority were under 10 base pairs (bps). Most of the indels were within coding regions, with the remainder in the gene regulatory regions. Although high confidence can be derived from the robust study design and meticulous human review, this extensive indel set has not undergone orthogonal validation. The extended benchmarking indel set, along with the indels in the previously published known-positive set, was the truth set used to benchmark indel calling pipelines in a community challenge hosted on the precisionFDA platform. This benchmarking indel set and reference samples can be utilized for a comprehensive evaluation of indel calling pipelines. Additionally, the insights and solutions obtained during the manual review process can aid in improving the performance of these pipelines.

## Introduction

Many genetic variants can have deleterious or outright oncogenic effects on gene function and disease susceptibility^1^. Indels (insertions and deletions) are a type of genetic variant that can disrupt the normal function of cancer related genes, leading to dampening of tumor suppressor pathways and/or the activation of oncogenic pathways^2^. Thus, an indel can drive uncontrolled cellular growth, leading to the formation of tumors^3^. In addition, indels can also lead to changes in protein structure and/or function, leading to further changes in cellular behavior that can contribute to the oncogenic process^4,5^. Therefore, accurate detection and characterization of indels is, a driving imperative not only for genetic research, but for the effective diagnosis, prognosis, and treatment of cancer^4,6^. Moreover, although gene editing technologies, such as CRISPR-Cas9, hold great promise for gene therapy^7^, off-target effects can lead to unintended indels^8^. Accurately detecting on- and off-target indels induced by gene editing is critical in evaluating their success. Variant identification methods need to be thoroughly evaluated to determine their sensitivity and specificity of indel detection to ensure the success of gene editing and the absence of unintended indels^9^.

Indels can be quite challenging to detect within the next-generation sequencing (NGS) data due to the variable length of indels and determining their proper alignment using often-short sequencing reads^10^. Accurately calling indels requires a combination of appropriate methods and techniques, including using specifically designed indel-calling algorithms, increasing the sequencing depth to improve the signal-to-noise ratio for the detection of low-frequency indels, and using multiple independent methods and filtering criteria to reduce the false positive rate (FPR). The utility of detected indels is increased by carefully assessing the indel calling performance and applying appropriate quality control measures, and annotating the indel calls with functional information for downstream analysis and interpretation of the results^11,12^.

Evaluating indel calling performance is an important step toward ensuring accuracy and identifying any potential issues or sources of error. This can be achieved by comparing the indel calling results to a benchmarking reference indel set (i.e., known set of indels) to assess the recall and precision of such indel calls^13^. Creating a large corpus of benchmarking indel sets for assessing the performance of indel calling results from different oncopanel sequencing platforms, different sequencing depths, and different sample types has been quite challenging^14^. The US Food and Drug Administration (FDA)-led Sequencing Quality Control Phase 2 (SEQC2) consortium made an essential contribution to the research community by identifying DNA variant content of a genomic reference sample (i.e., Sample A, a.k.a. the Universal Human DNA Reference)^15^. This sample was identified as having a high number of known positives (termed KnownPositives) including single nucleotide variants (SNVs), multi-nucleotide variants (MNVs), and small indels, as well as negatives within the consensus target region (CTR)^15^. Sample A was the primary reference sample for performance assessment for small to large oncopanels as well as liquid biopsy assays. To extend the applicability of the genomic reference sample in larger and cancer-focused gene regions, the SEQC2 Oncopanel Sequencing Working Group created an extended benchmarking indel set by an extensive manual curation review with the following strategies taken into consideration: (1) location ideally within the exon region of Catalogue of Somatic Mutations in Cancer (COSMIC) Cancer Gene Census^16^; (2) identified by multiple indel calling methods; (3) identified on multiple whole exome sequencing panels/DNA library duplicates; (4) reviewed by multiple reviewers and consistently identified as positive; and, as added confidence, (5) be reported in public databases. It is important to note that creating a known set of indels is time-consuming and can be prone to error, and it requires significant expertise and careful attention to detail, especially for indels with low variant allele frequency (VAF). Fortunately, by capitalizing on the design of the reference sample and utilizing a highly comprehensive sequencing dataset for the individual cell lines, this has facilitated the precise identification of indels with moderate VAF (primarily ranging between 10 and 30%) within individual cell lines. The SEQC2 Oncopanel Sequencing Working Group has successfully crafted a benchmark indels dataset of notable reliability through meticulous manual examination of NGS data. Although this study provides a high-confidence indel set with a robust study design, indels with low VAF may still need to be validated to ensure their accuracy.

## Results

### Study design

**Figure 1:**
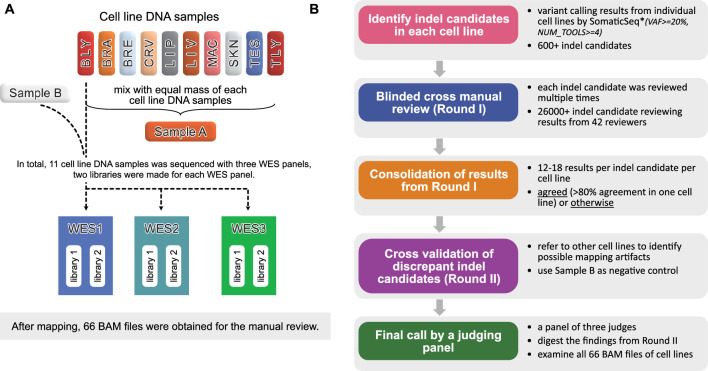
(**A**) Reference Sample A was created by mixing equal mass of DNA samples from 10 cancer cell lines, i.e., Myeloma (B-lymphocyte, BLY), Glioblastoma (brain, BRA), Adenocarcinoma (breast, BRE), Adenocarcinoma (cervix, CRV), Liposarcoma (soft tissue, LIP), Hepatoblastoma (liver, LIV), Lymphoma (macrophage, MAC), Melanoma (skin, SKN), Carcinoma (testes, TES), Carcinoma (T-lymphoblast, TLY). Sample B was DNA sample derived from a normal male control cell line. These 11 DNA samples were sequenced using three WES panels, i.e., Roche MedExome panel (WES1), IDT xGen Exome panel (WES2), and Agilent SureSelect Exome panel (WES3). For each WES panel, two library replicates were made and sequenced. In total, 66 BAM files were obtained after alignment to hg19 reference genome. (**B**) Overall block diagram of the manual review process in this study. The figure illustrates the main steps of the indel manual review process.

We utilized the whole exome sequencing (WES) datasets from our prior SEQC2 study for the manual review of indels. Briefly, the reference Sample A was created by mixing equal mass of DNA samples from 10 cancer cell lines. And the Sample B was DNA sample derived from a normal male control cell line. The DNA samples from these 11 cell lines were sequenced individually using three WES panels: WES1, WES2, and WES3 (see “Methods”). And for each WES panel, two library replicates were generated and sequenced, resulting in a total of 66 sequencing datasets for the study (Fig. 1A). This sample design enabled the identification of high confident indels in Sample A by reviewing indel candidates identified in individual cell lines with a high variant allele frequency (VAF). For example, if an indel is present in only one cell line with a VAF of 20%, its expected VAF in Sample A will be approximately 2%. To ensure the confident calling of indels, we established a review team to manually assess all indel candidates using individual cell line data. The manual review process involved five major steps (Fig. 1B): (1) identify indel candidates, (2) blinded cross-manual review (Round-I), (3) consolidation of results from Round-I, (4) cross-validation of discrepancy indel candidates (Round-II), and (5) final call by a panel of three judges.

Firstly, we identified indel candidates by filtering the variant calling results of individual cell lines from SomaticSeq (see “Methods”), taking the indels with a VAF >  = 20% and which were called with at least 4 callers (NUM_TOOLS >  = 4). Then we restricted the candidates to be within the exon regions of COSMIC Census genes. Thus, 604 indel candidates were selected for the manual review.

Secondly, we randomly assigned each indel candidate per cell line sequencing data to two or three reviewers, with the cell line sequencing information masked, so that the reviewers were not able to recognize which cell line they were reviewing and whether they were reviewing the same indel candidate. Review results were collected from 42 reviewers. "True", "False", "Ambiguous", or "N.A." was assigned per indel, per cell line, per whole exome sequencing (WES) panel, and per library replicate by reviewers.

Thirdly, we consolidated the results with a naïve criterion to take the conclusion from a majority (see “Methods”). The purpose of this manual review was to identify indels in Sample A. In total, we have 509 indel candidates considered to be "True", 20 indel candidates as "False", and 75 "Ambiguous" indel candidates that needed further review.

Next, we provided the 75 indel candidates that could not be resolved in the first round to the reviewers performing the second round of manual review. In this round, we sent the sequencing data of all cell lines, including Sample B as a control, WES panels, and library replicates to each reviewer, which enabled cross-validation of the "Ambiguous" indel candidates.

Finally, all the information collected from both rounds of review for the 75 "Ambiguous" indel candidates was reviewed and a final call was made by a panel of three expert judges at National Center for Toxicological Research (NCTR), FDA.

### Manual review results

In total, 516 "True" indels^17^ were identified in this indel manual review. 238 indels were located within the CTR, and 280 indels were outside CTR. In the KnownPositive set from our previous study, there were only 34 indels located within the COSMIC Census gene regions. Thus, this extended indel set came boosted the number of known indels for Sample A within COSMIC Census gene regions by a factor of 14x.

**Figure 2:**
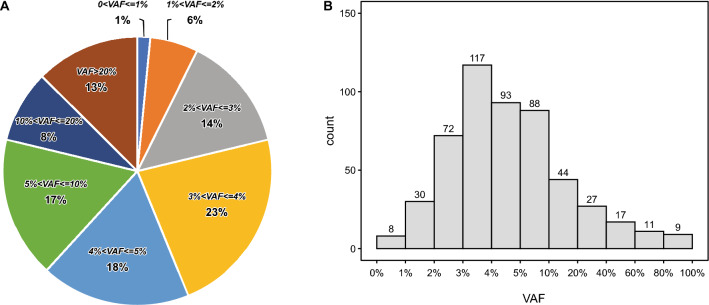
Indel VAF distribution. (**A**) Binned VAF distribution by percentage of indels. Each color represents a different VAF bin. (**B**) Number of indel variants found across the VAF spectrum of VAF. Bar height represents more the number of indel variants found within each VAF bin. VAF bins are represented on the X-axis and are non-linear.

We calculated the indel VAF based on WES sequencing data from Sample A and individual cell lines (“Methods”). Eighty-seven percent of indels (452) had a VAF less than or equal to 20% (Fig. 2). A large number of indels (62%) had a VAF in the 1–5% range, making the set suitable for benchmarking the indel calling for somatic variants.

**Figure 3:**
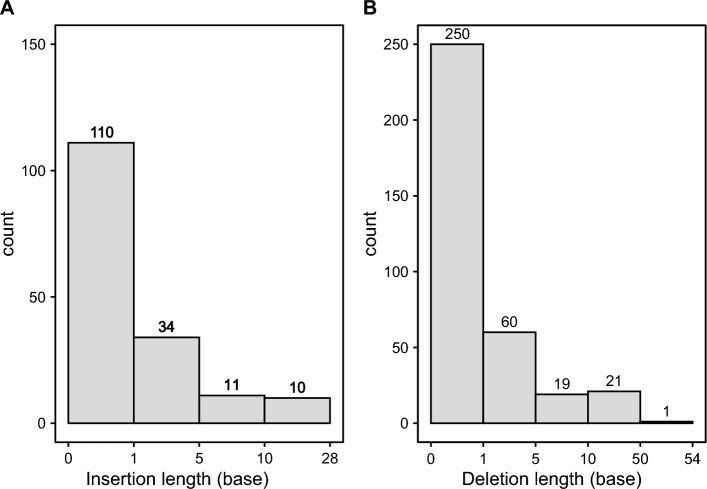
Length of insertions (**A**) and deletions (**B**). Bar height represents the number of indel variants found at in each that length. The x-axis represents the length of the genomic event, either insertion (**A**) or deletion (**B**).

The indel set contained 167 insertions and 351 deletions (Fig. 3). For the insertions, 67% (110) were 1 base, 20% (34) were 2–5 bps, 7% (11) were 6–10 bps, and 6% (10) were above 10 bps. The longest insertion in this dataset was 27 bps. For the deletions, 71% (250) were 1 base, 17% (60) were 2–5 bps, 5% (19) were 6–10 bps, and 6% (22) were above 10 bps. The longest deletion was 54 bps.

**Figure 4:**
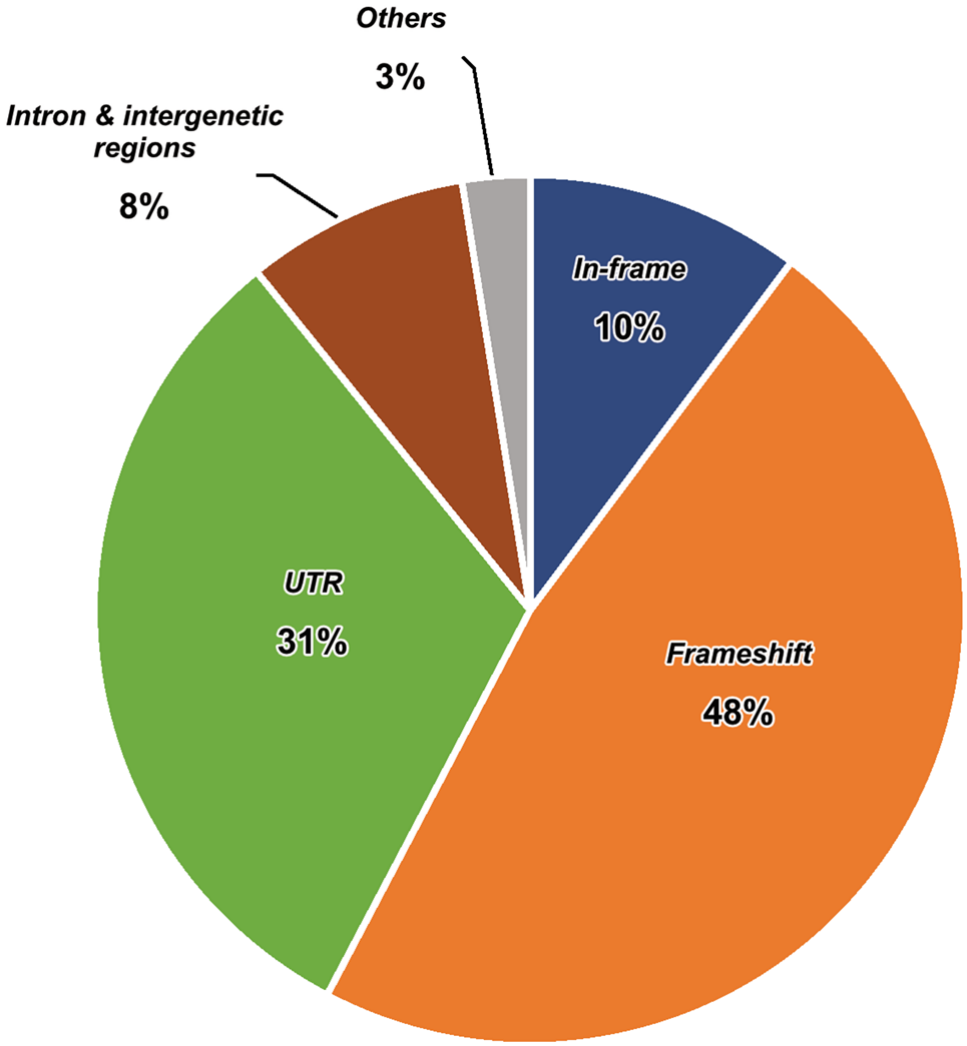
Genomic impact of indels, broken down into frameshift, UTR, in-frame, intron/intergenic region, and others.

Using annotation by snpEff, 10% (53) were in-frame indels (21 conservative and 32 disruptive), 48% (246) were frameshift indels, 31% (163) were in the UTR regions (43 in 5′-UTR and 120 in 3′-UTR), 8% (43) were in the intronic regions (23) and intergenic regions (20), and 3% (13) were other types (Fig. 4, Supplemental Table 1). Of note, due to genome annotation differences between the UCSC Genome Browser (UCSC Genes track, knownGene table) and databases used by snpEff (by default), some of the regions were annotated as "exon" in the UCSC Genome Browser but were annotated as "intron" or "intergenic regions" by snpEff (see “Methods”). We also labeled the indels with classes identified by RepeatMasker (see “Methods”). There are 53 indels in the repeat regions and users can conveniently exclude them from their analyses, particularly if indels within repeat regions do not align with their research objectives.

### Challenges in the manual review

During the manual review process, our team encountered several challenges in accurately calling indels with NGS data. A common issue was misaligned reads caused by repetitive regions, leading to false calls or incorrect VAF estimates. We found some of the alignments can be corrected with local realignment methods, but many cannot. Our reviewers used IGV to visualize read alignments and make visual adjustments based on their expertise and experience, ultimately rescuing indels that would have otherwise been missed.

Another challenge was the length of the indels. Due to the short read length of Illumina-based NGS, we had to limit the length of indels to no more than 50 bps (with only one exception of a 54-base deletion). Misalignment often occurred at the ends of reads, particularly for long insertions where only a few bps at each end were aligned with mismatches. This was also an issue in long deletions due to the large opening gap and allowance for mismatches.

Indel calling can be especially difficult in complex genomic regions such as highly repetitive content, segmental duplications, or structural variations. During the manual review, the team used RepeatMasker files from IGV and 1000 Genomes^18^ Phase 3 variants (v5c) to help pinpoint correct indels.

Another consideration was multi-allelic indels, which can sometimes be reported by a variant calling pipeline, particularly in repetitive regions. In some cases, one alternative allele may dominate, while the VAF of all alternative alleles was comparable in other cases. Our team compared all cell lines to determine whether the multiple alleles were true or due to random errors.

Sequencing errors introduced during library preparation and the sequencing platform can also lead to false positive calls. By comparing the six technical replicates (two library preparations for each of the three WES panels), we were able to identify most sequencing errors.

Lastly, the presence of other genomic variations, such as SNVs or structural variations, can impact the accuracy of indel calls. Our team found several indel candidates close to other SNVs and used manual alignment to verify the presence, position, and length of such candidates.

### Using the benchmarking indel set to evaluate the indel calling performance in the precisionFDA challenge

The benchmarking indel set was used to evaluate the indel calling performance in the precisionFDA "NCTR Indel Calling from Oncopanel Sequencing Data Challenge". Precision, recall, and F-1 score were calculated for valid challenge submissions using the benchmarking indel set along with the indels reported in the KnownPositive set in our previous study. The challenge was closed on July 26, 2022, and the award and final report were presented in the FDA/NCTR-MAQC 2022 Conference (5th annual meeting of the MAQC Society) at the U.S. FDA Headquarters on September 26, 2022. Please visit precisionFDA challenge websites^19,20^ for details about the challenge^21^.

## Conclusion

The benchmarking indel set represents a significant extension of the “KnownPositive variant set” for the benchmarking reference sample (i.e., Sample A) in our previous study, by adding 516 translationally relevant indels^17^ in COSMIC gene regions in addition to the already-reported 547 indels. The successful application of the benchmarking indel set in the precisionFDA challenge has proven the utility of performance evaluation of indel calling pipelines. Additionally, we have also obtained insights and solutions during the manual review process, which can be used to improve the performance of indel calling pipelines.

## Discussion

The value of a technology or process can only be determined when it is thoroughly assessed. Thus, to realize the value of indel calling to advance precision medicine, it is imperative to establish a method to inquire and rank various indel calling approaches as well as highlight their strengths and weaknesses. In the context of indel calling, a set of known variants is an absolute prerequisite to enable benchmarking or comparison of methods. Such known variants provide the "questions on the test" and thus must be of high quality and able to interrogate indel calling approaches from many different angles.

To this end, the SEQC2 consortium created a genomic reference sample with a high number of known positive indels (as well as other variants and negatives within the consensus target region (CTR)^15^. This work was published, and thus to create a fair and balanced "test", a new set of variants needed to be created for the precisionFDA indel calling challenge. To meet this goal, a team of highly expert reviewers was assembled to manually curate additional variants, with an emphasis on those relevant to precision oncology. A process was designed such that each variant was reviewed initially by 2 or 3 reviewers, and then variants which were not agreed upon were subject to a deeper round of review with six reviewers before going to internal NCTR experts.

The resulting additional indel call set indeed spans many different VAFs, lengths, locations, and kinds of indel types. Importantly, for any indel calling pipeline to have an application in precision medicine, it must be able to perform in several contexts (VAF ranges, genomic context, etc.) and not just one specific scenario. This is equivalent to having questions spanning many topics on a test rather than just focusing on one area.

The reference samples and the true indel sets, especially the indels that are associated with COSMIC genes, can be very useful in assessing the accuracy and effectiveness of gene editing technologies. By processing the reference samples in parallel to target gene editing samples, one can evaluate the efficiency and precision of the gene editing technologies. This will indicate either shortcomings in detecting off-target effects (allowing the technologies to be further optimized) or promote trust in the proper assessment of off-target effects, which will advance gene editing technologies toward clinical application.

This study does have certain key limitations. While a very thorough manual review was performed, indels were neither not independently confirmed using an orthogonal technology. Moreover, this study focused only on certain genomic regions, and many cancer-related genes and variants may be later discovered. Despite these limitations, this study does have additional benefits beyond merely creating a data resource and enabling the precisionFDA challenge. In addition to creating a community-based available resource of an indel known reference set, this study has also resulted in defining a clear process that could be leveraged for other kinds of genomic variants or even in other disease contexts to create a rigorous manually curated known dataset. Hg38 (GRCh38) is a more recent and enhanced assembly compared to hg19, with continuous updates to the human genome reference. In our study, the decision to use hg19 as the reference genome was guided by the prevalent use of hg19 in real-world clinical practices. Many routine clinical workflows and databases have been established on the foundation of hg19, prompting us to align our study with these prevailing standards. It's important to note that the indel set presented in this study represents its inaugural version. We intend to stay abreast of advancements in the human genome reference and plan to update the indel set in the future, ensuring its relevance and accuracy within the evolving landscape of genomic research and clinical applications.

Using multiple aligners and different reference genome releases may help to identify some possible false positives by contrasting the misalignment bias of various aligners and reference genome releases. Such practice could provide additional information to the reviewers. In this study, we addressed this potential misalignment bias problem by extensive manual local realignment carried out carefully by the reviewers. Knowing that the community will eventually adopting hg38 or an even newer reference genome release, like Telomere-to-Telomere (T2T)^22^, we will be working on a future update release of the indel true set to complement the current release for hg19.

## Methods

### Identification of indel candidates

In this study, we utilized three whole exome sequencing (WES) data sets, namely WES1, WES2, and WES3, which were originally generated as part of our prior SEQC2 study^15^. As an artificial reference sample, Sample A is a mixture of DNA samples of 10 Agilent Universal Human Reference (UHR)^23^ cell lines. No experimental animals or human participants were involved in the study. A total of 66 BAM files resulting from aligning the raw FASTQ files to the hg19 reference genome were used for the manual review. Specifically, two library replicates were made for each of the 10 cancer cell lines (which made up the Sample A in our previous SEQC2 study^15^) and Sample B^15^ and the libraries were sequenced with three WES panels on Illumina sequencing platforms and produced 66 FASTQ files (22 files for each WES panel). All libraries and sequencing data passed the quality control. Variant calling results (in VCF format) from SomaticSeq^24^ were collected for all technical replicates for internal use of the SEQC2 Oncopanel Sequencing Working Group (Supplemental Fig. 1). For each VCF result, non-SNV variants were taken if VAF ≥ 20% and called with at least four callers. To avoid missing genuine indels, such as those with lower variant allele frequencies near the 20% threshold, we opted to include indel candidates present in any of the two replicates of a cell line. All these variants were then pooled together and filtered kept if any part of a variant was located within the CTR regions or the COSMIC Census gene regions and the UCSC exon regions. The COSMIC Census gene list includes genes that are frequently mutated in cancer, and knowledge of indels in these genes can be fuel for diagnosing cancer, determining prognosis, and/or pinpointing a precision medicine course of treatment of cancer patients. After excluding the indels that are already in the KnownPositive set, we collected a set of indel candidates for the manual review.

### Recruitment of reviewers and data distribution

Members of the SEQC2 Oncopanel Sequencing Working Group as well as other expert researchers in the members' research teams were invited to volunteer for the indel manual review. Forty-two reviewers from 15 institutes undertook the first round of manual review. Eight reviewers from seven institutes also performed the second round of the manual review. A judging panel of three researchers at NCTR was formed to make final decisions. Two advisers were invited to share their knowledge and experiences with the reviewers and provided helpful guidance for ambiguous indel candidates. All reviewers and judges had agreed not to participate in the precisionFDA "NCTR Indel Calling from Oncopanel Sequencing Data Challenge" to avoid conflict of interest.

Indel candidates were split into groups containing about 100 candidates in each group. Each technical replicate data of each indel candidate group was randomly assigned to two to three reviewers (two if the candidates were reported in the 1000 Genomes Phase 3 variants, three otherwise) to review. The meta information was removed from the BAM files, and smaller BAM files containing the reads around the indel candidates of each group were created for easier distribution. All filenames were masked with random strings. Files were copied and named differently when the files were sent to different reviewers. The judging panel created a master table for proper tracking back (Supplemental Table 3). Reviewers were assigned a reasonable workload to ensure a high-quality thorough manual review.

### Tools and rules

Manual review is a time-consuming process that requires significant expertise and attention to detail. Knowledge and experience have been shared and questions and concerns have been discussed across the reviewers. IGV^25^ was used for visualizing the BAM files, and IGVNav (https://github.com/griffithlab/igvnav) was used for quick navigation among indel candidates and marking issues and making comments. Some basic rules were applied in the manual review:

- The exchange of data and reviewing of other results were prohibited to keep the review result independent.
- Manually align the reads to the reference genome when needed.
- RepeatMasker files from IGV and 1000 Genomes Phase 3 variants (v5c) were supplied to the reviewers for reference.
- Any issues that were found in the review process should be reported by either marking with flags or writing comments with the IGVNav app. The information was collected and digested for the second round of manual review and the final judging.
- Cross -validation among cell lines was performed in the second round of manual review and cross -validation among technical replicates as well as the cell lines were performed by the judging panel to make final calls.

### Consolidation of results from Round-I review

After we collected all the results from the reviewers in the first round of manual review, we consolidated the results with naïve criteria. For every indel candidate in each cell line, we collected 12–18 reviewing results. We considered an indel candidate in a specific cell line to be "True" if more than 80% of reviewing results were reported as "True", and an indel candidate to be "False" if more than 80% of reviewing results were reported as “False”. If an indel candidate was “True” in at least one cell line, the candidate was considered as “True” in Sample A. If an indel candidate was “False” in all cell lines that have been reviewed for this candidate, it was considered as “False” in Sample A. All other indel candidates were considered as “Ambiguous” in Sample A. An example is shown in Supplemental Table 4. Thus, 509 indel candidates were "True", 20 indel candidates were "False", and 75 were "Ambiguous", according to the naïve criteria.

### Round-II: cross validation of indel candidates of discrepancy review results

In the second round of manual review, each of the 75 "Ambiguous" indel candidates were reviewed by six reviewers. Each reviewer examined one of the three WES sequencing data of all 10 cell lines, which enabled them to refer to other cell lines to determine whether the candidate is an artifact or a true one. Two other reviewers examined all six sequencing datasets of Sample B and reported any findings in the Sample B, which served as a reference for the judge panel to make decisions. An example is shown in Supplemental Table 5.

### Final call by a panel of three judges

A judge panel of three researchers was formed to make final calls decisions on the "Ambiguous" indel candidates, by examining all WES panel data for all cell lines, as well as Sample A and Sample B, and taking all the information available to them into consideration, including but not limited to: the results and reasons/comments from Round I & II reviewers, the coverage, frequency, sequence context, and manually reads realignment.

### Annotation of the indels

The indels were annotated with SnpEff^26^ (version 5.0e) using the default settings for hg19. Although we intended to select the indel candidates within the exon regions, the databases which SnpEff used for annotation may have different annotation compared with UCSC database, which we used for the indel candidate selection step. Thus, there is 11% of the indels were annotated to locate outside exon regions by the SnpEff.

We also labeled the indels with the information from RepeatMasker^27^ (with repeat masking data sourced from IGV), by adding “RepeatMasker = [class]” in the info section of the vcf file. If an indel is not located in any repeat regions reported by RepeatMasker, “RepeatMasker = None” is added. Otherwise, the “[class]” could be any or combination of “DNA”, “LINE”, “LTR”, “Low_complexity”, “RC”, “RNA”, “SINE”, “Satellite”, “Simple_repeat”, or “Other”. These classes are defined by RepeatMasker as documented in UCSC GenomeBrowser (https://genome.ucsc.edu/cgi-bin/hgTrackUi?g=rmsk):

- DNA: DNA repeat elements;
- LINE: long interspersed nuclear elements;
- LTR: long terminal repeat elements, which include retroposons;
- Low_complexity: low complexity repeats;
- RC: rolling circle;
- RNA: RNA repeats (including RNA, tRNA, rRNA, snRNA, scRNA, srpRNA);
- SINE: short interspersed nuclear elements (SINE), which include ALUs;
- Satellite: satellite repeats;
- Simple_repeat: simple repeats (micro-satellites);
- Other: other repeats.

### Supplementary Information

Supplementary Information.

## Data Availability

The benchmarking data set^17^ of 516 indels in VCF format and the associated reporting regions (hg19) in BED format are available at figshare (10.6084/m9.figshare.24183801).
