# Supplementary material for: Extend the benchmarking indel set by manual review using the individual cell line sequencing data from the Sequencing Quality Control 2 (SEQC2) project
Source: Sci Rep. 2024 Mar 25;14:7028. doi: 10.1038/s41598-024-57439-7 (PMC10963753; doi:10.1038/s41598-024-57439-7)
Supplement: Supplementary file 1 — Supplementary Information. [file 41598_2024_57439_MOESM1_ESM.pdf]

Extend the Benchmarking Indel Set by Manual Review Using the Individual Cell line Sequencing Data from the Sequencing Quality Control 2 (SEQC2) project

Binsheng Gong[1], Dan Li[1], Yifan Zhang[1], Rebecca Kusko[2], Samir Lababidi[3], Zehui Cao[4], Mingyang Chen[5], Ning Chen[6], Qiaochu Chen[4], Qingwang Chen[4], Jiacheng Dai[5], Qiang Gan[7], Yuechen Gao[4], Mingkun Guo[8], Gunjan Hariani[9], Yujie He[8], Wanwan Hou[4], He Jiang[4], Garima Kushwaha[10], Jian-Liang Li[11], Jianying Li[11], Yulan Li[12], Liang-Chun Liu[7], Ruimei Liu[4], Shiming Liu[13], Edwin Meriaux[14], Mengqing Mo[15], Mathew Moore[16], Tyler J. Moss[17], Quanne Niu[4], Ananddeep Patel[18], Luyao Ren[4], Nedda F. Saremi[19], Erfei Shang[4], Jun Shang[4], Ping Song[20], Siqi Sun[16], Brent J. Urban[18], Danke Wang[5], Shangzi Wang[21], Zhining Wen[8], Xiangyi Xiong[12], Jingcheng Yang[4], Lihui Yin[22], Chao Zhang[4], Ruolan Zhang[4], Ambica Bhandari[16], Wanshi Cai[6], Agda Karina Eterovic[18], Dalila B. Megherbi[14], Tieliu Shi[13], Chen Suo[15], Ying Yu[4], Yuanting Zheng[4], Natalia Novoradovskaya[19], Renee L. Sears[23], Leming Shi[4], Wendell Jones[9], Weida Tong[1] & Joshua Xu[1*]

[1] Division of Bioinformatics and Biostatistics, National Center for Toxicological Research, U.S. Food and Drug Administration, Jefferson, AR 72079, USA
[2] Cellino Bio, 750 Main Street, Cambridge, MA 02143, USA
[3] Office of Data Analytics and Research, Office of Digital Transformation, Office of the Commissioner, US Food and Drug Administration, Silver Spring, MD 20993, USA
[4] State Key Laboratory of Genetic Engineering, School of Life Sciences and Human Phenome Institute, Fudan University, Shanghai 200438, China
[5] Human Phenome Institute, Fudan University, Shanghai 201203, China
[6] iGeneTech Bioscience Co., Ltd., 8 Shengmingyuan Rd., Changping, Beijing, China
[7] Clinical Diagnostics Division, Thermo Fisher Scientific, 46500 Kato Rd., Fremont, CA 94538, USA
[8] College of Chemistry, Sichuan University, Chengdu, Sichuan 610064, China
[9] Q^2 Solutions, 2400 Ellis Road, Durham, NC 27703, USA
[10] Guardant Health, Inc., 505 Penobscot Drive, Redwood City, CA 94063, USA
[11] Integrative Bioinformatics Support Group, National Institute of Environmental Health Sciences, National Institutes of Health, Research Triangle Park, NC 27709, USA
[12] College of Life Sciences, Shanghai Normal University, Shanghai 200234, China
[13] Center for Bioinformatics and Computational Biology, and the Institute of Biomedical Sciences, School of Life Sciences, East China Normal University, Shanghai 200241, China
[14] CMINDS Research Center, University of Massachusetts, Lowell, MA 01854, USA
[15] Department of Epidemiology, School of Public Health, Fudan University, Shanghai 200032, China
[16] ResearchDx, Irvine, CA 92618, USA
[17] Eurofins Viracor, LLC, 18000 W 99th St., Lenexa, KS 66219, USA
[18] Eurofins Viracor Biopharma Services, Inc., 18000 W 99th St., Lenexa, KS 66219, USA
[19] Agilent Technologies, Inc., 11011 N Torrey Pines Rd., La Jolla, CA 92037, USA
[20] Cancer Genomics Laboratory, Department of Genomic Medicine, MD Anderson Cancer Center, Houston, TX 77030, USA
[21] State Key Laboratory of Genetic Engineering and Collaborative Innovation Center for Genetics and Development, School of Life Sciences, Fudan University, Shanghai 200438, China
[22] PathGroup, Nashville, TN 37217, USA
[23] Velsera, 6 Cityplace Dr Suite 550, Creve Coeur, MO 63141, USA

[*]Corresponding author(s): Joshua Xu (Joshua.Xu@fda.hhs.gov)

Supplementary Figures

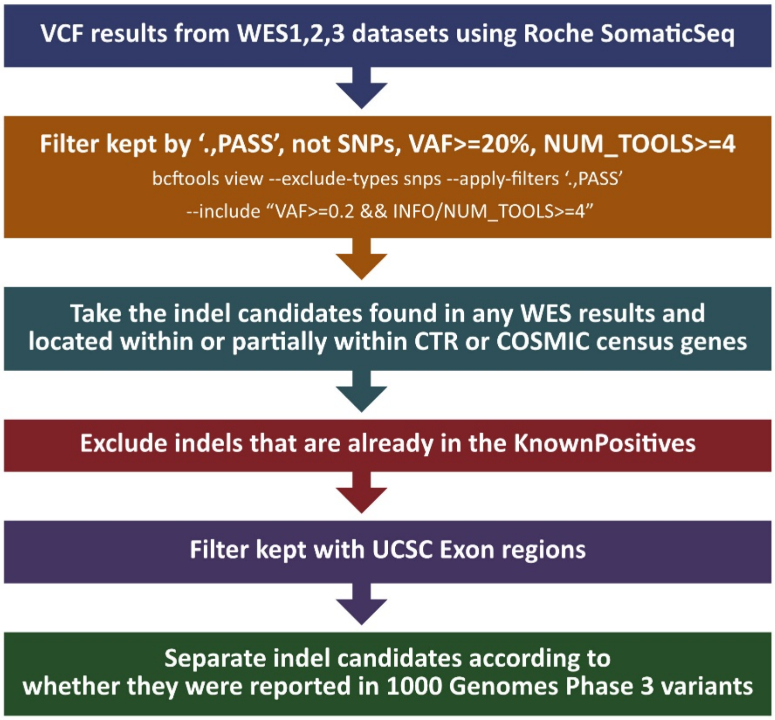

Supplemental Figure 1: Schematic of data flow processing and filtering to arrive at the final known set. Indel candidates were then split into two groups based on whether they were reported in the 1000 Genomes Phase 3 variants (v5c). Supplemental Table 2 shows the number of indel candidates in each cell line and Sample B, and the numbers of indel candidates that were reported in the 1000 Genomes Phase 3 variants.

Supplementary Tables

Supplemental Table 1: Numbers of indels in different categories

**Frameshift**		**246**
	frameshift_variant	232
	frameshift_variant&missense_variant	1
	frameshift_variant&splice_acceptor_variant&splice_region_variant&intron_variant	1
	frameshift_variant&splice_donor_variant&splice_region_variant&intron_variant	1
	frameshift_variant&splice_region_variant	4
	frameshift_variant&start_lost	3
	frameshift_variant&stop_gained	2
	frameshift_variant&stop_lost	2
**In-frame**		**53**
	conservative_inframe_deletion	11
	conservative_inframe_deletion&splice_region_variant	1
	conservative_inframe_insertion	7
	start_lost&conservative_inframe_deletion	1
	stop_gained&conservative_inframe_insertion	1
	disruptive_inframe_deletion	22
	disruptive_inframe_insertion	10
**UTR**		**161**
	3_prime_UTR_variant	119
	5_prime_UTR_variant	42
**Intron**		**23**
	intron_variant	23
**Intergenic regions**		**20**
	upstream_gene_variant	11
	downstream_gene_variant	9
**Others**		**13**
	splice_acceptor_variant&intron_variant	1
	splice_acceptor_variant&splice_donor_variant&intron_variant	1
	splice_donor_variant&conservative_inframe_deletion&splice_region_variant&intron_variant	2
	splice_region_variant&intron_variant	6
	non_coding_transcript_exon_variant	2
	bidirectional_gene_fusion	1

Supplemental Table 2: For each cell line, the number of indel candidates vs. those reported in 1000 Genomes Phase 3.

CELL LINE	Indel candidates	Reported in 1000 genomes phase 3 sites
**B**	87	55
**CL01**	77	48
**CL02**	78	59
**CL03**	79	51
**CL04**	80	55
**CL05**	84	59
**CL06**	89	59
**CL07**	95	59
**CL08**	99	71
**CL09**	331	64
**CL10**	387	96
**Unique**	604	208

Supplemental Table 3: Example data distribution. Reviewers were given random identifiers, and each sub-dataset had 2-3 reviewers. The total number of variants sent to any individual reviewer was limited to ensure focus and high quality. File names were deidentified to provide a truly blinded review.

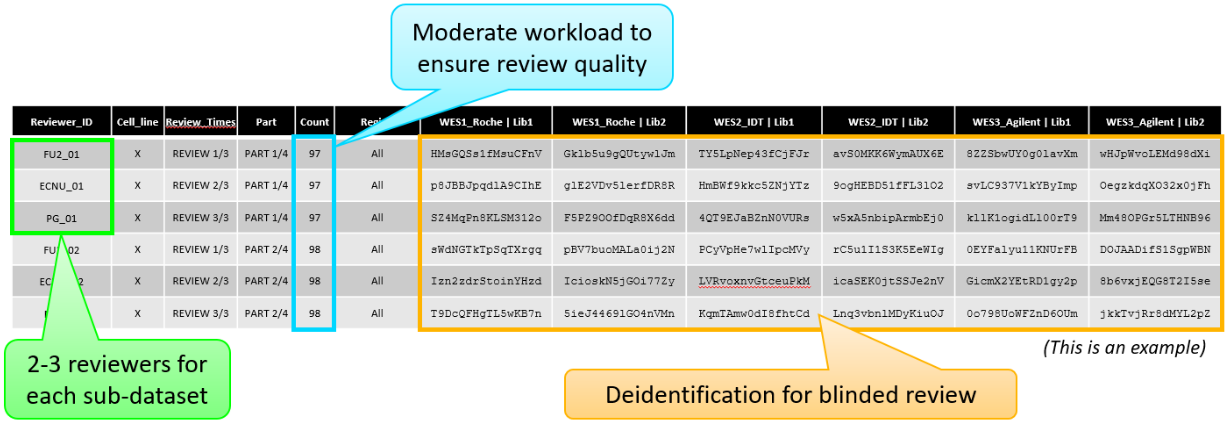

Reviewer_ID	Cell_line	Review_Times	Part	Count	Regi[illegible]	WES1_Roche | Lib1	WES1_Roche | Lib2	WES2_IDT | Lib1	WES2_IDT | Lib2	WES3_Agilent | Lib1	WES3_Agilent | Lib2
FU2_01	X	REVIEW 1/3	PART 1/4	97	All	HMsGQSs1fMsuCFnV	Gklb5u9gQUtywlJm	TY5LpNep43fCjFJr	avS0MKK6WymAUX6E	8ZZSbwUY0g0lavXm	wHJpWvoLEMd98dXi
ECNU_01	X	REVIEW 2/3	PART 1/4	97	All	p8JBBJpqdlA9CIhE	glE2VDv5lerfDR8R	HmBWf9kkc5ZNjYTz	9ogHEBD51fFL31O2	svLC937V1kYByImp	OegzkdqXO32x0jFh
PG_01	X	REVIEW 3/3	PART 1/4	97	All	SZ4MqPn8KLSM312o	F5PZ9OOfDqR8X6dd	4QT9EJaBZnN0VURs	w5xA5nbipArmbEj0	kllK1ogidLl00rT9	Mm48OPGr5LTHNB96
FU[illegible]02	X	REVIEW 1/3	PART 2/4	98	All	sWdNGTkTpSqTXrgq	pBV7buoMALa0ij2N	PCyVpHe7wlIpcMVy	rC5u1I1S3K5EeWIg	0EYFalyu11KNUrFB	DOJAADifS1SgpWBN
EC[illegible]2	X	REVIEW 2/3	PART 2/4	98	All	Izn2zdrStoinYHzd	IcioskN5jGOi77Zy	LVRvoxnvGtceuPkM	icaSEK0jtSSJe2nV	GicmX2YEtRD1gy2p	8b6vxjEQG8T2I5se
[illegible]	X	REVIEW 3/3	PART 2/4	98	All	T9DcQFHgTL5wKB7n	5ieJ44691GO4nVMn	KqmTAmw0dI8fhtCd	Lnq3vbnlMDyKiuOJ	0o798UoWFZnD6OUm	jkkTvjRr8dMYL2pZ

(This is an example)

Supplemental Table 4: Example of True (T), False (F), and Ambiguous (A) calls for variants

VariantID	Cell-Line	WES	Lib	Call
chr1_40367597_40367598_G_GCC	X	WES1_Roche	Lib1	F
chr1_40367597_40367598_G_GCC	X	WES1_Roche	Lib1	F
chr1_40367597_40367598_G_GCC	X	WES1_Roche	Lib1	F
chr1_40367597_40367598_G_GCC	X	WES1_Roche	Lib2	F
chr1_40367597_40367598_G_GCC	X	WES1_Roche	Lib2	F
chr1_40367597_40367598_G_GCC	X	WES1_Roche	Lib2	F
chr1_40367597_40367598_G_GCC	X	WES2_IDT	Lib2	A
chr1_40367597_40367598_G_GCC	X	WES2_IDT	Lib2	A
chr1_40367597_40367598_G_GCC	X	WES2_IDT	Lib2	T
chr1_40367597_40367598_G_GCC	X	WES2_IDT	Lib1	T
chr1_40367597_40367598_G_GCC	X	WES2_IDT	Lib1	T
chr1_40367597_40367598_G_GCC	X	WES2_IDT	Lib1	T
chr1_40367597_40367598_G_GCC	X	WES3_Agilent	Lib2	A
chr1_40367597_40367598_G_GCC	X	WES3_Agilent	Lib2	A
chr1_40367597_40367598_G_GCC	X	WES3_Agilent	Lib2	A
chr1_40367597_40367598_G_GCC	X	WES3_Agilent	Lib1	T
chr1_40367597_40367598_G_GCC	X	WES3_Agilent	Lib1	A
chr1_40367597_40367598_G_GCC	X	WES3_Agilent	Lib1	A

Supplemental Table 5: Example of originally "Ambiguous" variants where the call in all cell lines was compared to Sample B to help arrive at the final call.

variant_ID	Final call	Call in cell-lines	Call in Sample B
chr1_27107262_27107264_TC_T	F	F:6	F
chr6_33286387_33286388_C_CTT	F	F:6	F
chr22_36708120_36708134_ATCAGCTTTGTCCT_A	T	T:6	F
chr11_3721949_3721955_ACAGTG_A	T	T:6	F
chr5_86672844_86672845_A_ATAAG	T	T:6	F
chr11_22646886_22646892_AGATTT_A	T	T:6	F
